# Role of Foreign-Born Status on Suicide Mortality in Spain Between 2000 and 2019: An Age-Period-Cohort Analysis

**DOI:** 10.3389/ijph.2022.1604538

**Published:** 2022-05-18

**Authors:** Gonzalo Martínez-Alés, Catherine Gimbrone, Caroline Rutherford, Katherine Keyes, Teresa López-Cuadrado

**Affiliations:** ^1^ Department of Epidemiology, Mailman School of Public Health, Columbia University, New York, NY, United States; ^2^ Centro de Investigación Biomédica en Red de Salud Mental (CIBERSAM), Madrid, Spain; ^3^ Instituto de Investigacion Hospital Universitario La Paz (IdiPAZ), Madrid, Spain; ^4^ National Centre of Epidemiology, Carlos III Health Institute (ISCIII), Madrid, Spain

**Keywords:** social determinants of health, epidemiological model, migrant health, suicide, age-period-cohort modelling

## Abstract

**Objectives:** To examine recent age-period-cohort effects on suicide among foreign-born individuals, a particularly vulnerable sociodemographic group in Spain.

**Methods:** Using 2000–2019 mortality data from Spain’s National Institute of Statistics, we estimated age-period-cohort effects on suicide mortality, stratified by foreign-born status (native- vs. foreign-born) and, among the foreign-born, by Spanish citizenship status, a proxy for greater socioeconomic stability.

**Results:** Annual suicide mortality rates were lower among foreign- than native-born individuals. There was heterogeneity in age-period-cohort effects between study groups. After 2010, suicide mortality increased markedly among the foreign-born—especially for female cohorts born around 1950, and slightly among native-born women—especially among female cohorts born after the 1960s. Among native-born men, suicide increased linearly with age and remained stable over time. Increases in suicide among the foreign-born were driven by increases among individuals without Spanish citizenship—especially among cohorts born after 1975.

**Conclusion:** After 2010, suicide in Spain increased markedly among foreign-born individuals and slightly among native-born women, suggesting an association between the downstream effects of the 2008 economic recession and increases in suicide mortality among socioeconomically vulnerable populations.

## Introduction

Suicide is a major contributor to global mortality and the leading cause of violent death [1]. Suicide deaths generate significant emotional impact on families and communities and important consequences for society. Reducing suicide mortality is an urgent public health need.

Characterizing temporal variations in suicide mortality rates is important for public health and healthcare planning as well as to guide the generation of new causal hypotheses. While global suicide mortality has decreased over the last 3 decades, largely due to marked decreases in suicide in China and India [2], trends in suicide rates vary across locations – for instance, they have increased markedly in specific countries, such as the United States [3].

Spain has traditionally had one of the lowest suicide mortality rates across Europe and among high-income countries [4]. There is, however, generalized concern that suicide mortality rates may have increased in Spain over the last decade, in the context of the aftermath of the 2008 great economic recession, as major economic downturns [5] and especially increases in unemployment [6] are generally associated with increases in suicide rates. Research has shown that suicide mortality increased following the 2008 great economic recession in most European countries, with particularly salient increases in male suicides [6–9]. Although Spain was one of the hardest hit European countries during and after the 2008 great economic recession, with a 300% increase in unemployment rates between 2007 and 2013 [10], whether there was a contemporaneous increase in suicide mortality has been largely debated in the literature and remains unclear [11–17]: While an initial interrupted time series analysis suggested an upward deviation in Spain’s suicide mortality trends following onset of the economic downturn [11], and a following analysis indicated moderate increases in suicide only among women [15], the most recent evidence indicates that overall age-standardized suicide rates remained roughly stable between 2004 and 2018 in Spain [17].

Age, period, and cohort effects can provide critical illumination into the patterns, risk groups, and potential causes of suicide mortality trends [18–27]. Age effects result from factors that are specific to developmental stages, period effects result from factors that impact individuals across age and birth cohort, and cohort effects result from factors that individuals born into a specific context share over the life course [28]. Age, period, and cohort effects have important public health and clinical implications, as they can guide identification of potential actionable mechanisms for prevention. Recent age-period-cohort analyses of Spain’s 1984–2018 suicide mortality data did not identify increasing period or birth cohort effects over the last 2 decades for the general population [17].

Examining suicide trends among specific sociodemographic groups is also critical for suicide prevention efforts as it allows for early identification and targeting of emerging high-risk populations. For instance, evidence indicates that recent increases in suicide in the United States were driven by surges in suicide rates among racially minoritized youth [27]. The apparent lack of variation in suicide trends in Spain after the 2008 economic recession may have hidden increases in suicide rates among specific sociodemographic groups, particularly vulnerable to the effects of economic downturns. In Spain, foreign-born status is an important marker of socioeconomic and racial/ethnic minoritization in the general population. Following the 2008 great economic recession, foreign-born individuals living in Spain experienced harder increases in unemployment rates than native-born counterparts [29]. In addition, foreign-born individuals were targeted by specific austerity measures, such as the “Real Decreto Ley 16/2012” law [30], based on which Spain’s universal health coverage was interrupted between 2012 and 2018, and non-urgent and specialized medical care became restricted for foreign-born individuals without legal permanent residence permit. The overall negative health effects of the 2008 great economic recession on Spain’s foreign-born population have been reported in the literature [31]. The objective of this study was to estimate the age-period-cohort effects underlying 2000–2019 trends in suicide in Spain, focusing on the role of foreign-born status. We examined differences between foreign-born individuals with and without Spanish citizenship—a proxy for access to permanent residence permit, longer time since migration, greater socioeconomic stability, and stronger social support networks, to better understand the potential role of social vulnerability.

## Methods

### Data Source

We obtained 2000–2019 mortality data from Spain’s National Institute of Statistics [32]. These data are based on Spain’s National Mortality Registry, a single cause-of-death mortality database, and consist of International Classification of Disease, Tenth Revision (ICD-10) codes based on the underlying cause of death as indicated by medical examiners in death certificates. Deaths were designed as attributable to suicide using the following ICD-10 codes for underlying cause of death: X60-X84, and Y87.0, following widely adopted practices. We also retrieved information on sex (male/female), age in years, foreign-born status (foreign-born/native-born), and Spanish citizenship status (yes/no) for each death. We designated deceased individuals as foreign-born if they were born outside of Spain and resided in Spain, regardless of Spanish citizenship. Denominator data, used for the calculation of rates, were also obtained from the National Institute of Statistics’ Ongoing Register of Residents [33]. Suicide deaths of individuals not residing in Spain were excluded from rate calculations.

### Analyses

#### Descriptive Analyses

All study procedures were conducted separately for foreign- and native-born suicides. We began by generating an age-period contingency table where data were separated into age groups and periods, both grouped in 5-year intervals. Then, we described suicide mortality rates by age, period, and cohort, using traditional two-dimensional graphical representations. These representations are informative for initial examination of the data as summarized in the initial contingency table, as they provide the initial evidence of presence of period or cohort effects. Two-dimensional plots can represent age variations in suicide rate across periods or cohorts, cohort variations across age or periods, and period variations across age or cohorts [34]. In addition, we implemented descriptive hexagonal grids, where each data point (i.e., each age-specific suicide rate at a specific year period, and thereby the corresponding birth cohort-specific suicide rate at a specific year period, as Cohort = Period-Age) is represented using a hexagonal piece [35]. By simultaneously representing age, period, and cohort-specific suicide rates, hexagonal grids allow for an intuitive visual interpretation, overcoming limitations of traditional two-dimensional graphical representations. Descriptive analyses were conducted using Stata 16 and R 3.6.2 with R Studio 1.4.1717.

#### Age-Period-Cohort Analyses

Next, we modeled age, period, and cohort effects. To overcome overidentification problems due to linear dependance (as Cohort = Period-Age) [28], we used methods based on the approach developed by Clayton and Schiffer [36]. First, we estimated a categorical age predictor of suicide mortality rates over time. Second, we introduced a “drift” parameter, which is the sum of the linear period and cohort effects over time. Third, we estimated first and second derivatives of the “drift” parameter and regressed them on period and cohort and attributed them to specific periods and cohorts, in order to estimate the extent to which suicide mortality trends accelerated or decelerated for each period and cohort. Fourth, we calculated relative rates for each period- and cohort- specific deviation from of the “drift” parameter, using 2010 as reference period. At each stage, we assessed model fit by including age + “drift” compared to age alone and then iteratively adding period and cohort effects, examining whether model fit improved with parameters addition, and iteratively removing each parameter, examining whether model fit worsened following parameter removal. Age-period-cohort modelling was conducted using the “apc.fit” function from the R package “Epi”.

#### Sensitivity Analyses

As a first sensitivity analysis, we obtained stratified age-period-cohort effect estimates for suicide mortality for foreign-born individuals with and without Spanish citizenship, a proxy for permanent residence permit, given that only foreign-born people with permanent residence permit could access specialized healthcare and welfare services for a large proportion of the study period, due to specific austerity measures. Also, like permanent residence permit, Spanish citizenship is usually granted several years after migrating to Spain [37], and immigrants who have resided in a host country for a longer period tend to have more socioeconomic stability and stronger social and familial support networks, two important correlates of suicide mortality risk. Data on Spanish citizenship was only available for the 2003–2019 period.

In addition, we implemented an alternative approach to age-period-cohort modelling, based on the multi-phase method [34], to test the extent to which our estimates were robust to model misspecification. For this method, we 1) log-transformed suicide mortality rates per age group and period, 2) conducted a median polish analysis by removing the log-additive effect of age (row) and period (column) by iteratively subtracting the median value of each row or column until the row and column medians approximated zero, 3) plotted the residuals by cohort, 4) and assessed cohort effects by conducting a linear regression where residuals were regressed on cohort category – exponentiation of a cohort’s beta parameter yields an excess suicide rate attributable to each cohort that can be compared to the referent cohort. We repeated this procedure to obtain age and period effects. Multi-phase age-period-cohort modelling was conducted using Stata 16.

## Results

Between 2000 and 2019, there were 68,549 deaths by suicide in Spain, for the following annual suicide rates: 13.8 per 100,000 men, 4.2 per 100,000 women, and 8.9 per 100,000 people overall. Among native-born people, annual suicide rates were as follows: 14.4 per 100,000 men, 4.3 per 100,000 women, and 9.2 per 100,000 people overall. Among foreign-born people, annual suicide rates were as follows: 9.10 per 100,000 men, 3.2 per 100,000 women, and 6.2 per 100,000 people overall.

### Descriptive Analyses

**FIGURE 1:**
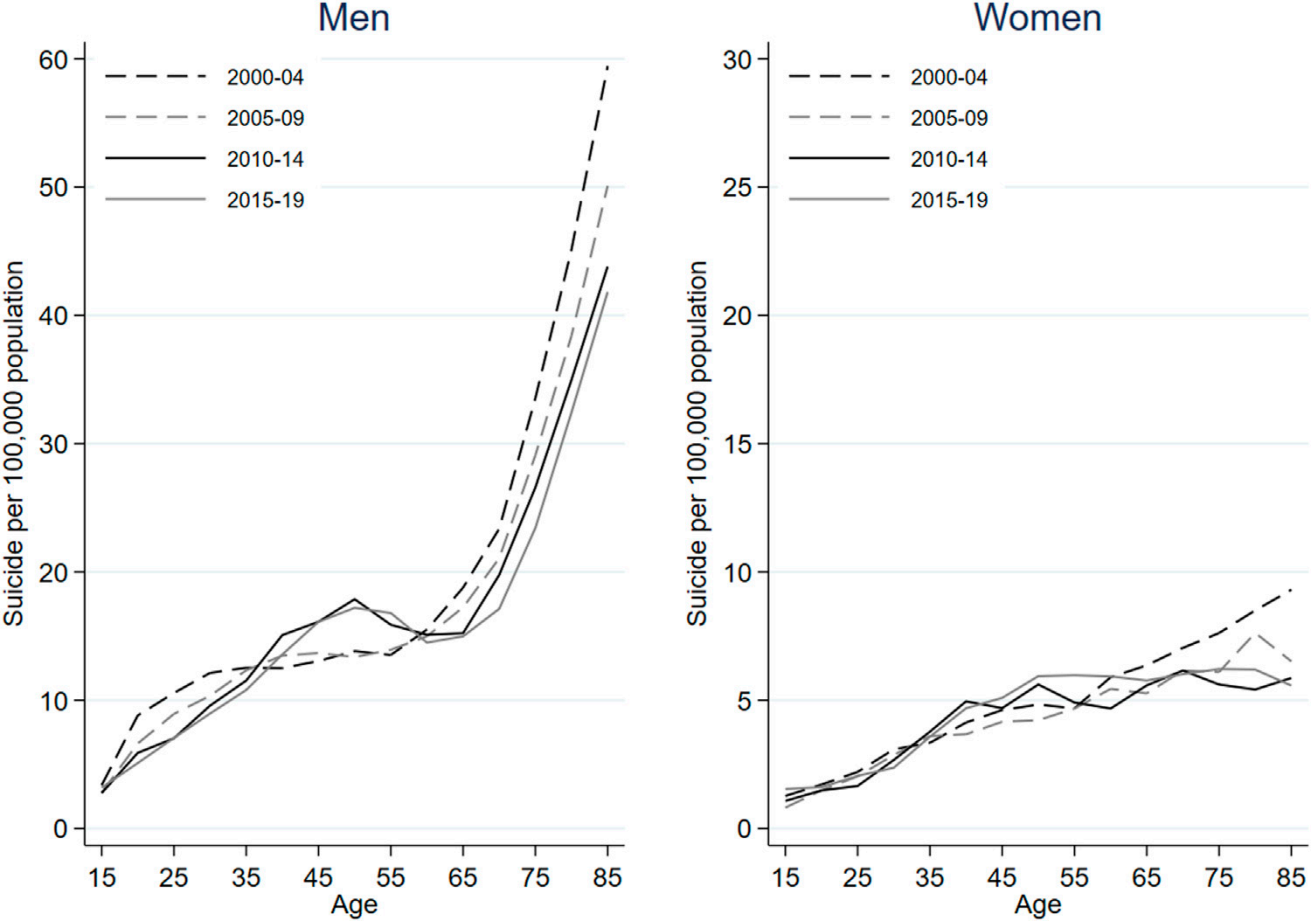
Period-specific suicide mortality rates across age among native-born males and females between 2000 and 2019 in Spain.

Figure 1 represents variations in period-specific suicide mortality rates across age among native-born individuals in Spain between 2000 and 2019, stratified by gender. Supplementary Figure S1 shows variations in age-specific suicide rates over time among native-born individuals in Spain between 2000 and 2019, stratified by gender. Supplementary Figure S2 displays the rate of suicide among native-born individuals in Spain between 2000 and 2019, stratified by gender, across age, period, and cohort. Suicide rates for each birth cohort can be visualized along the diagonal “C” isolines, with corresponding ages and periods along “A” and “P” isolines, respectively. Suicide rates among the native-born increased with age, especially among men, and remained stable or decreased over time except for individuals aged 40–54, for whom rates went up after 2010.

**FIGURE 2:**
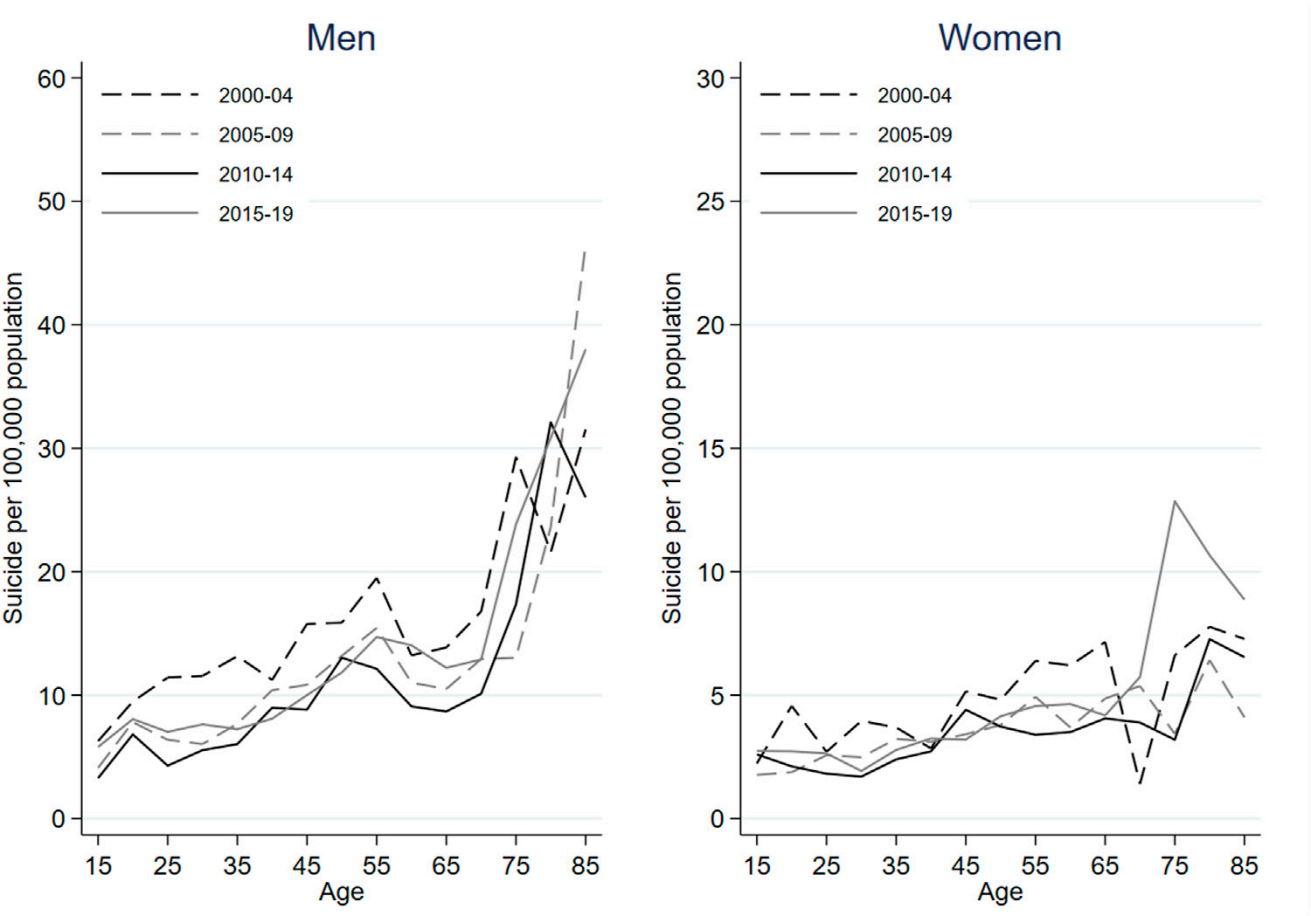
Period-specific suicide mortality rates across age among foreign-born males and females between 2000 and 2019 in Spain.

Figure 2 represents variations in period-specific suicide mortality rates across age among foreign-born individuals in Spain between 2000 and 2019, stratified by gender. Supplementary Figure S3 shows variations in age-specific suicide rates over time among foreign-born individuals in Spain between 2000 and 2019, stratified by gender. Notably, lines representing period-specific suicide rates over time are more heterogeneous and intersect more frequently in foreign-born than native-born suicides. Supplementary Figure S4 displays the rate of suicide among foreign-born individuals in Spain between 2000 and 2019, stratified by gender, across age, period, and cohort. Again, age effects are also evident, especially among men. Suicide rates among the foreign-born increased after 2010 across age groups except in men aged 40–54, with most notable increases taking place among older women.

### Age-Period-Cohort Models

Supplementary Tables S1, S2 provide the model fit statistics for overall age, period, and cohort contributions to native- and foreign-born suicide rates. Including age, period, and cohort parameters all improved model fit, and removing them all reduced model fit, indicating that all three parameters are sufficiently predictive of variance to be included in a final model.

**FIGURE 3:**
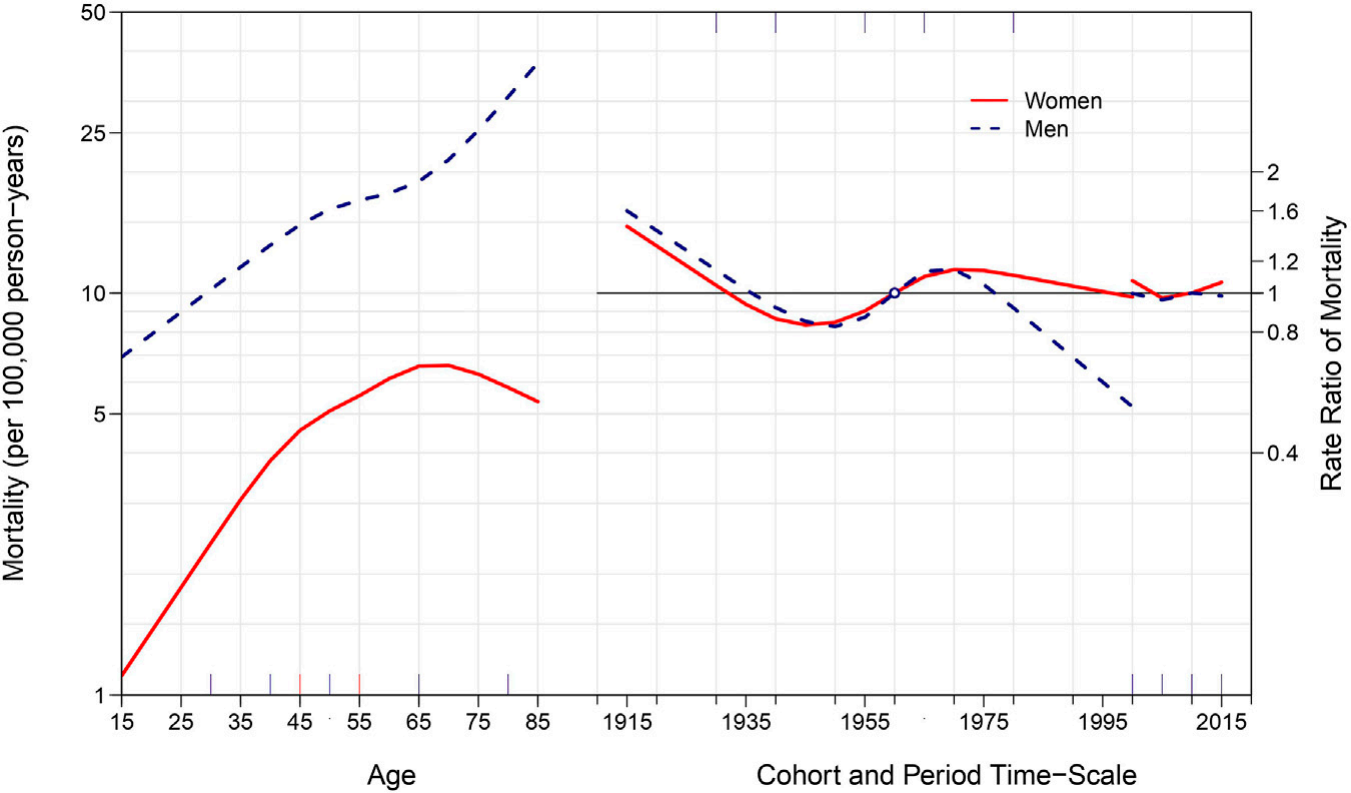
Age, period, and cohort effects on suicide among native-born males and females between 2000 and 2019 in Spain.

Figure 3 estimates age, period, and cohort effects in suicide mortality among native-born individuals residing in Spain between 2000 and 2019. In the left axis, the age effect is expressed as suicide rate per 100,000 person-years, anchored to the reference cohort (1960). Among native-born men, suicide mortality increased roughly linearly with age. Among native-born women, suicide mortality increased rapidly through adolescence and early and mid-adulthood, peaking at around age 60, and declining very slightly through late life. In the right axis, rate ratios allow for the comparison of each period and cohort with the reference period (2010) and cohort (1960). Among native-born men, suicide risk increased for birth cohorts born between 1950 and 1965 and decreased markedly for cohorts born thereafter. There was no evidence of a period effect. Among native-born women, suicide rates also increased in cohorts born between 1950 and 1970 and, as opposed to male cohorts, remained heightened thereafter, decreasing only slightly in birth cohorts born between 1975 and 2000. Period effects indicate that native-born female suicide rates decreased between 2000 and 2005 but have increased slightly since 2010.

**FIGURE 4:**
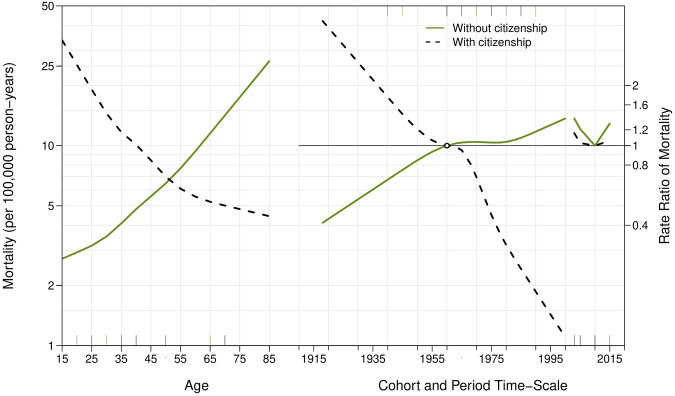
Age, period, and cohort effects on suicide among foreign-born males and females between 2000 and 2019 in Spain.

Figure 4 represents age, period, and cohort effects in suicide mortality among foreign-born individuals residing in Spain between 2000 and 2019. While suicide rates increased markedly among foreign-born men after age 45, age effects were clearer among foreign-born females, with starker increases in suicide rates across middle and late lives. Among foreign-born men, suicide rates decreased continuously across birth cohorts. Among cohorts of foreign-born women, suicide initially increased, peaking among women born in 1950, and subsequently decreased in younger cohorts. There were clear period effects for foreign-born men and women, indicating initial decreases in suicide rates during the early 2000s, followed by increases after 2010.

**FIGURE 5:**
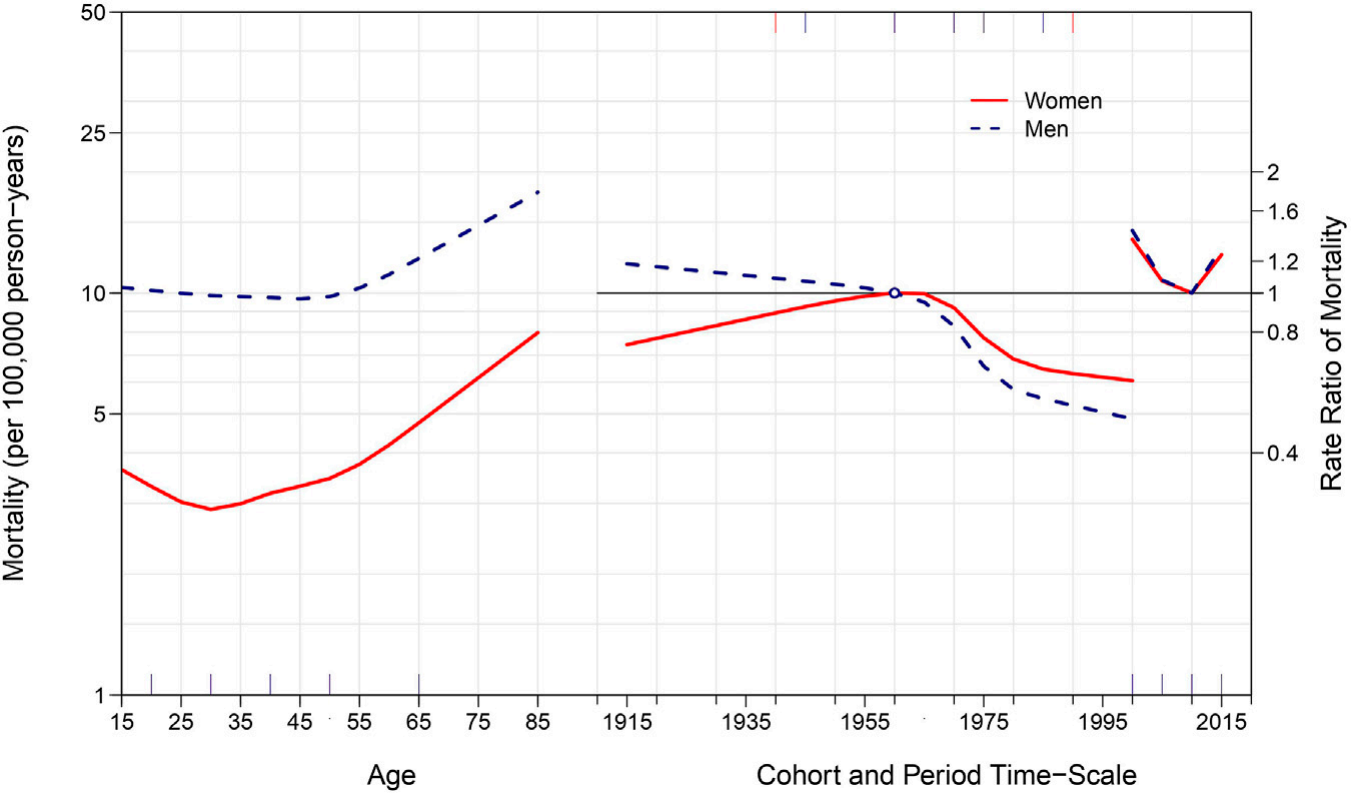
Age, period, and cohort effects on suicide among foreign-born individuals between 2000 and 2019 in Spain, stratified by Spanish citizenship status.

Figure 5 represents age, period, and cohort effects on suicide rates among foreign-born individuals with and without Spanish citizenship, respectively. Results indicate that suicides among foreign-born individuals without citizenship increased markedly among recent birth cohorts and, across age groups and birth cohorts, after 2010. Supplementary Figures S5, S6 show age-period-cohort effect estimates among native-born and foreign-born people, respectively, based on the multi-phase method: interpretation of the results did not change. Supplementary Figures S7–S9, show age-period-cohort effect estimates among native- and foreign-born individuals and after stratification foreign-born suicides by citizenship status, respectively, obtained using a different cohort reference year (1935): interpretation of the results did not change.

## Discussion

Between 2000 and 2019 in Spain, foreign-born individuals had overall lower suicide mortality rates than native-born counterparts. Age-period-cohort models, however, revealed that suicide mortality increased markedly after 2010 among foreign-born individuals, with a specific peak among foreign-born females born around 1950; and slightly among native-born women—especially among female cohorts born after the 1960s. Meanwhile, suicide rates remained roughly stable for native-born men, decreasing for cohorts born after the 1960s. To the best of our knowledge, this is the first study to examine the age-period-cohort effects-underlying recent suicide trends among migrants to a Southern European country after the 2008 great economic recession. Our results highlight that foreign-born individuals and native-born women—especially young adult female cohorts, are emerging high-risk groups for suicide in Spain.

Our finding that recent increases in suicide mortality rates among foreign-born people living in Spain were largely driven by period effects (i.e., external factors circumscribed to a specific temporal moment that manifest in changing rates across age groups and birth cohorts) highlights the potential role of downstream consequences of the 2008 great economic recession. There is long-standing evidence that the negative social and health effects of major economic downturns disproportionately impact migrants [29, 38]. Substantial attention has been directed towards the mediating role of unemployment, an important risk factor for suicide [39, 40], in suicide rate increases during and after economic crises [41]. In Spain, following the great recession, the unemployment rate went from 8.7% in 2005 to 17.9% in 2010 and 22.1% in 2012 among native-born individuals; and from 14.7% in 2005 to 30.5% in 2010 and 36.8% in 2012 among the foreign-born. Moreover, recovery during the aftermath of the economic recession also was heterogeneous between both sociodemographic groups: in 2019, unemployment affected 13.8% native-born and 20.8% foreign-born people residing in Spain [10]. It is important to mention that suicide risk is particularly high among people experiencing long-term unemployment [42].

There are additional stressors brought about by the great economic recession whose role was more salient among foreign- than native-born people residing in Spain. First, between 2010 and 2015, foreign-born individuals residing in Spain migrated massively to their original countries as well as to countries with less adverse socioeconomical conditions [29], conditioning a negative migratory balance and contributing to the socioeconomic and emotional erosion of their families and communities. Second, as mentioned, austerity measures adopted following the onset of the 2008 economic recession included the interruption of universal health coverage for immigrants without residency permit, leading to major negative health impacts on Spain’s foreign-born population [31]. Results from our sensitivity analyses focusing on immigrants without Spanish citizenship, a proxy category to identify individuals with reduced access to healthcare and welfare systems and at higher risk of social and economic deprivation, indicate that recent increases in suicide among foreign-born individuals were almost entirely driven by increases among the individuals without Spanish citizenship. Notably, in addition to period effects, we found remarkable increases in foreign-born individuals without citizenship born after 1975.

These results have important implications in terms of etiology as well as for clinical and public health stakeholders. First, recent increases in suicide risk among the foreign-born in Spain seem partially explained by trends in broader threats to the health of minoritized communities which were worsened by the great economic recession, its aftermath, and the subsequently adopted austerity policies. Avoiding or reversing austerity policies and expanding access to the welfare state to groups at risk of social and economic exclusion and minoritization, such as foreign-born individuals without Spanish citizenship, seems a straightforward solution to reduce the overall health impacts of economic recessions and, in particular, their effect on suicide mortality. In fact, the role of short- and long-term unemployment on suicide risk can be buffered by generous unemployment protection and overall expanded access to welfare [41]. Second, by characterizing a specific high-risk population group, our findings enhance suicide prevention efforts, especially in terms of identifying unmet needs regarding development and implementation of interventions. In addition to making healthcare universally available, enhancing immigrants’ access and engagement with healthcare, regardless of age and birth cohort, through deployment of cultural adaptive programs [43] that ensure culturally- and structurally-competent [44], easy to access mental healthcare and suicide-specific interventions is critical.

We characterized birth cohort and period effects in suicide mortality rates among native-born women, hence expanding previous work by Cayuela et al. [15] that suggested a moderate 2000–2016 increase in suicide risk among women in Spain. This is in line with recent evidence indicating that, while population-based estimates of the prevalence of depression may be decreasing in Europe, they are on the rise among Spanish young and middle-aged women [45] and with a previous population-based study that identified young women as particularly at risk for suicidal ideation and behaviors [46]. In addition, previous research has identified the great recession as a root cause of recent increases in mental health problems among the Spanish population [47]. There also were gender differences in the impact of the aftermath of the 2008 economic recession: while the initial years of economic recession reduced the gender gap in unemployment (the unemployment rate went from 7.8% in 2005 to 23.9% in 2012 among men, and from 13.5% in 2005 to 24.5% in 2012 among women), employment recovery was markedly slower for women, with 2018 rates sitting at 15.1% for men and 18.5% for women. Importantly, adolescents and young adults were the hardest hit demographic group, with 2012 unemployment rates of 71.4% and 47.7% for individuals aged 16–19 and 20–24 years respectively. While the gender gap in unemployment is reduced during crises, overall increases in part-time and precarious work ultimately re-establish women as a family dependent and flexible labor supply, increasing their socioeconomic vulnerability [48]. Accordingly, the combination of high and long-lasting unemployment rates among young women with other medium- and long-term gendered effects of the 2008 economic recession may partially explain these period and birth cohort effects.

Whether suicide rates increased in Spain during the aftermath of the great economic recession has been extensively debated in the literature [11–17]. While most recent research found no evidence of an overall increase, this was largely because suicide mortality rates in Spain are mostly driven by rates among native-born men and, as shown in our results, suicide mortality has remained stable in this sociodemographic groups since 2000. We found, however, marked age effects among native-born men, with remarkably high suicide rates in individuals aged 65 years and older. This finding is common in most Western cultures and is seemingly explained by elders experiencing barriers in access to mental healthcare during crises due to internalized stigma, social disconnectedness [49], and physical impairment [50], their higher rates of chronic medical comorbidity and disability, and their use of more lethal suicide methods than younger counterparts [51]. Despite this, suicide prevention interventions are typically targeted at other groups at risk, such as people with mental disorders or adolescents. Developing, implementing and scaling up age-friendly interventions to favor elders’ access to prevention efforts during crises is a largely unmet clinical and public health need [51, 52].

This study has limitations. First, our data on suicide mortality are subject to potential errors in suicide mortality certification [53–55]. Second, as with any age-period-cohort study, where regression identification issues make models vulnerable to potential model misspecification problems, validity of our results is dependent on appropriateness of modelling choices. However, we implemented two different approaches to age-period-cohort modelling and, in addition, conducted sensitivity analyses varying reference periods and cohorts, obtaining similar results that suggest robustness. Third, we cannot rule out that the post-2010 negative migratory balance may have altered the overall make-up of the migrant population group in Spain (e.g., driving a higher proportion of migrants at high suicide risk, such as those moving from areas with high suicide rates), partially explaining our results. Fourth, we lacked information on important correlates of vulnerability, especially as regards to foreign-born individuals (such as time since migration, legal residence permit, or socioeconomic status). While we were able to analyze foreign-born suicides separately by Spanish citizenship status, a proxy for permanent residence permit and hence for socioeconomic stability, social and family support networks, and access to specialized healthcare and social welfare, future research should incorporate data on additional social covariables to further advance understanding of recent trends in suicide in Spain.

In conclusion, suicide rates increased among native-born females and, especially, among foreign-born individuals after 2010. Suicide increases among foreign-born people were entirely driven by increases among individuals without Spanish citizenship—which were particularly stark in birth cohorts born after 1975. These results highlight the importance of examining suicide rates among especially vulnerable sociodemographic groups and lend support for an association between the downstream effects of the 2008 economic recession and an increase in suicide rates in foreign-born individuals living in Spain, who experienced the starkest increases in unemployment following onset of the recession and also were subject to specific austerity measures limiting access to specialized healthcare and welfare services.

## Data Availability

Data are publicly available from Spain’s Institute of Statistics.
