# Supplementary material for: Role of Foreign-Born Status on Suicide Mortality in Spain Between 2000 and 2019: An Age-Period-Cohort Analysis
Source: Int J Public Health. 2022 May 18;67:1604538. doi: 10.3389/ijph.2022.1604538 (PMC9156625; doi:10.3389/ijph.2022.1604538)

Age-period-cohort effects on suicide mortality in Spain between 2000-2019: moderation by migrant status - Supplementary material

Supplementary Table S1. Model fit statistics for age-period-cohort model for suicide rates among native-born individuals in Spain from 2000 to 2019 (Ref Cohort: 1960)

Model parameter	Change in deviance (degrees of freedom)		
	All	Male	Female
Age	-	-	-
Age-drift	55,5 (1)***	89,1 (1)***	1,3 (1)*
Age-cohort	519,2 (3)***	477,3 (3)***	103,9 (3)***
Age-period-cohort	24 (2)***	11,8 (2)***	25,3 (2)***
Age-period	-513,1 (-3)***	-475,5 (-3)***	-96,1 (-3)***
Age-drift	-30,1 (-2)***	-13,6 (-2)***	-33,1 (-2)***

*** p < .001, ** p < .01, * p < .05

Supplementary Table S2. Model fit statistics for age-period-cohort model for suicide rates among foreign-born individuals in Spain from 2000 to 2019 (cohort ref: 1960)

Model parameter	Change in deviance (degrees of freedom)		
	All	Male	Female
Age	-	-	-
Age-drift	51,3 (1)***	40,5 (1)***	3,4 (1)
Age-cohort	32,5 (3)***	22,8 (3)***	7,1 (3)
Age-period-cohort	120,7 (2)***	80 (2)***	19,3 (2)***
Age-period	-60,7 (-3)***	-20,5 (-3)***	-7,1 (-3)
Age-drift	-92,5 (-2)***	-82,4 (-2)***	-19,4 (-2)***

*** p < .001, ** p < .01, * p < .05

Supplementary Figure S1. Age group-specific suicide mortality rates over time among native-born males and females between 2000-2019 in Spain.

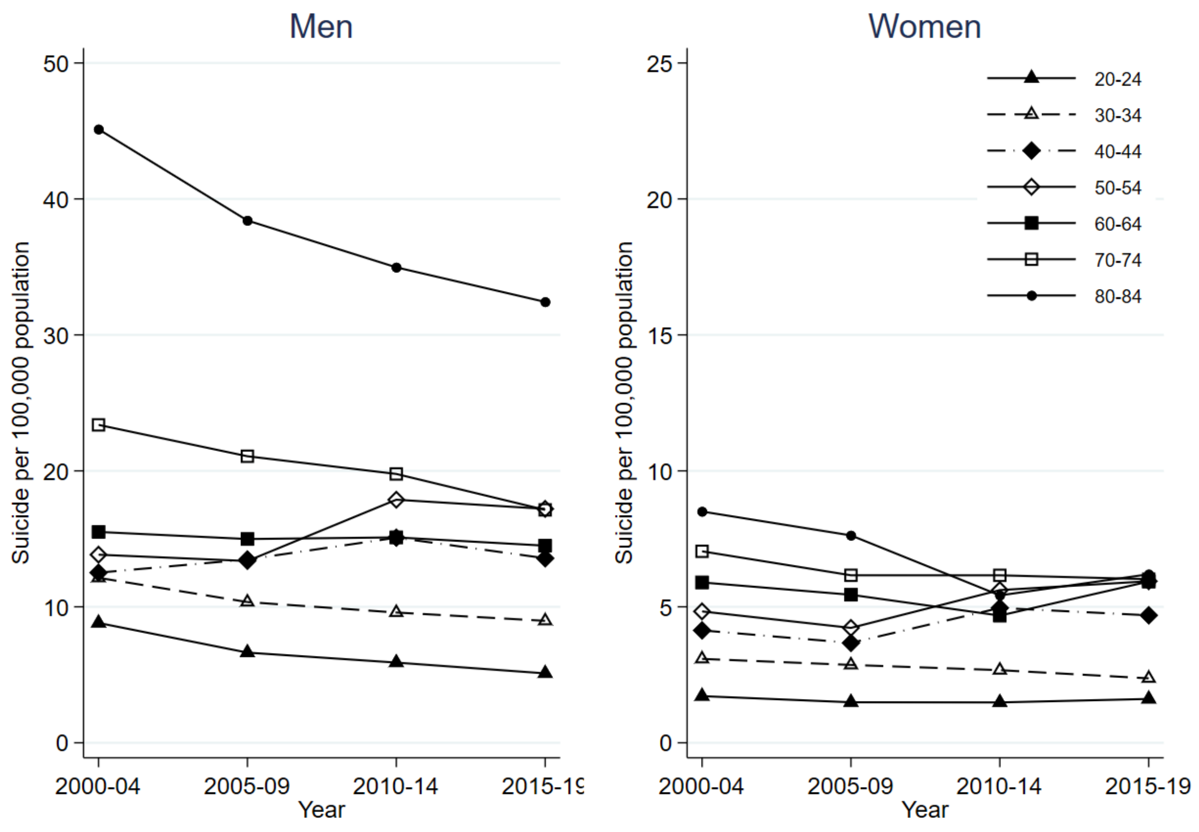

Supplementary Figure S2. Suicide mortality rates among native-born males and females between 2000-2019 in Spain across age, period, and cohort.

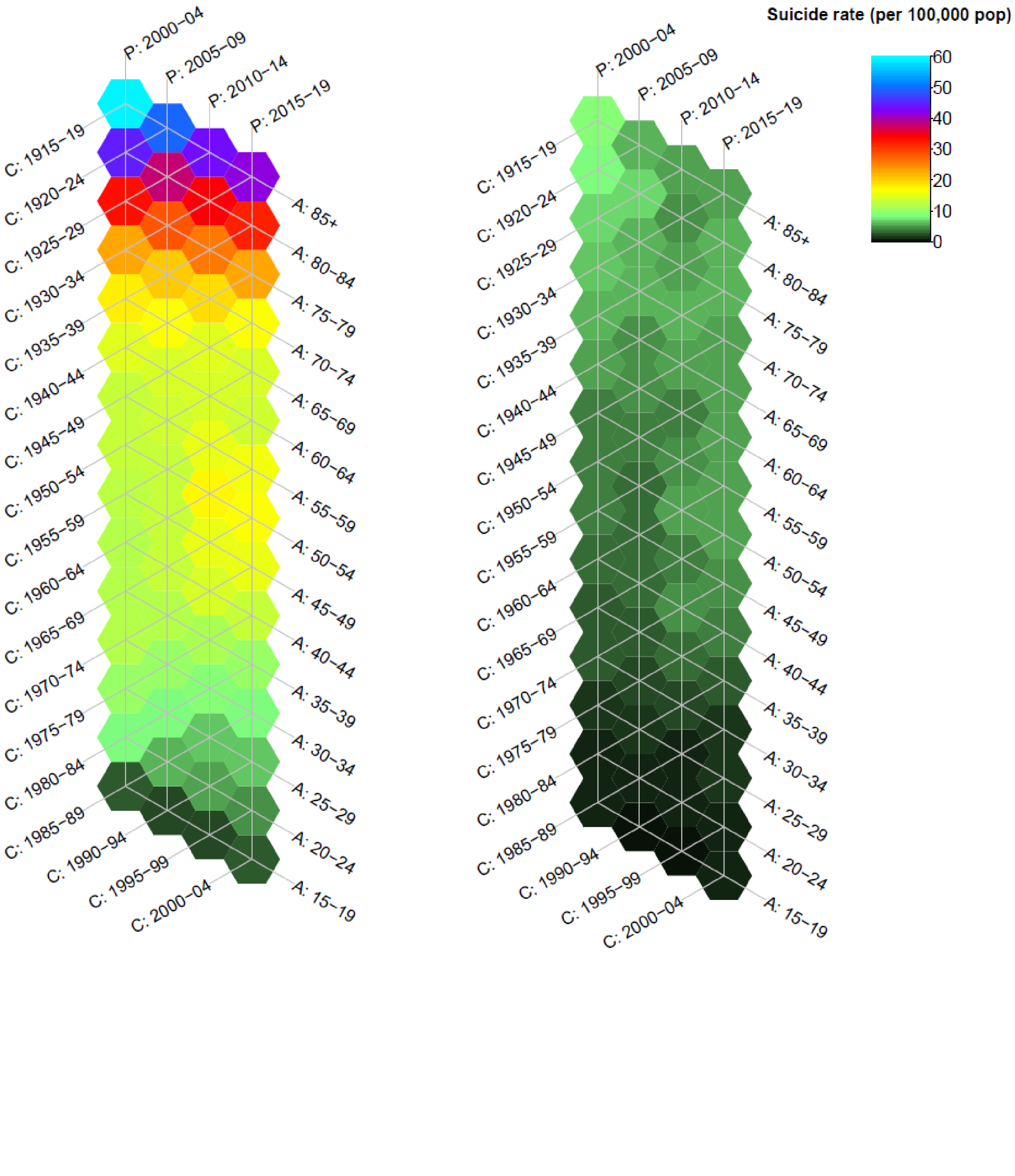

Supplementary Figure S3. Age group-specific suicide mortality rates over time among foreign-born males and females between 2000-2019 in Spain.

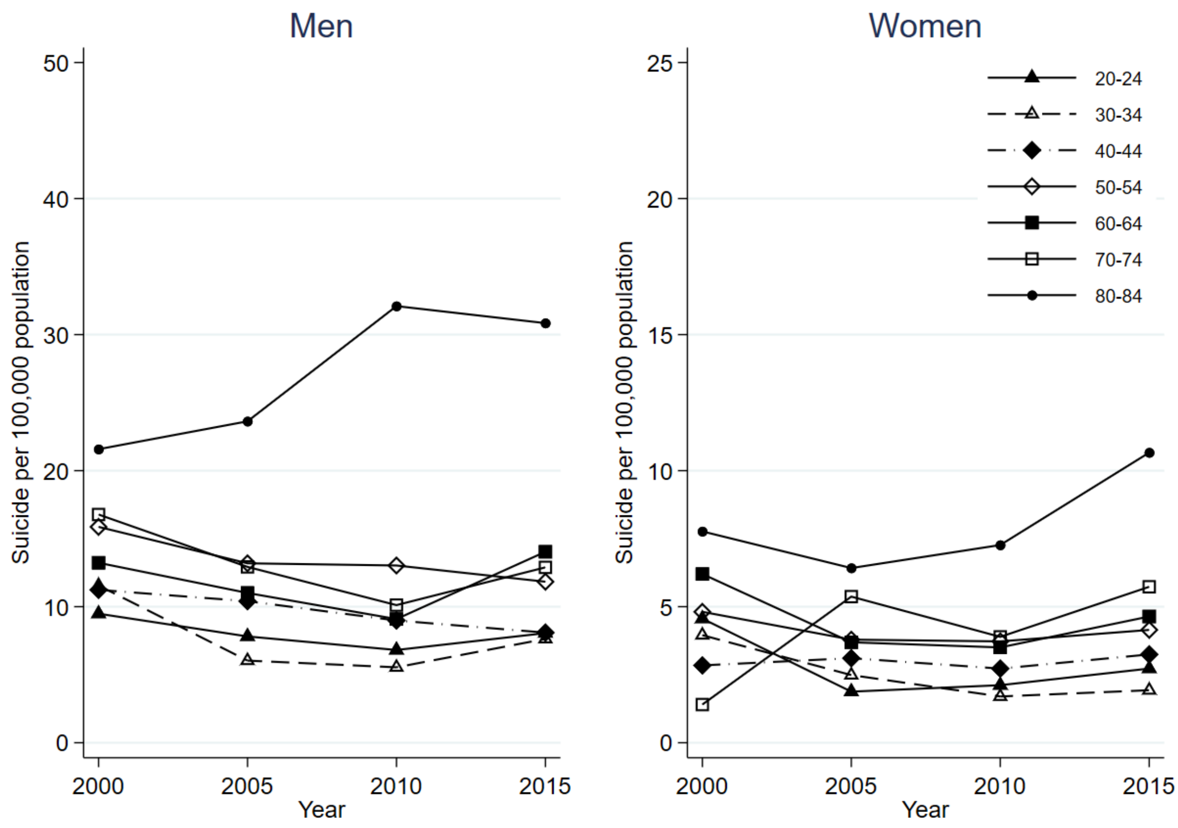

Supplementary Figure S4. Suicide mortality rates among foreign-born males and females between 2000-2019 in Spain across age, period, and cohort.

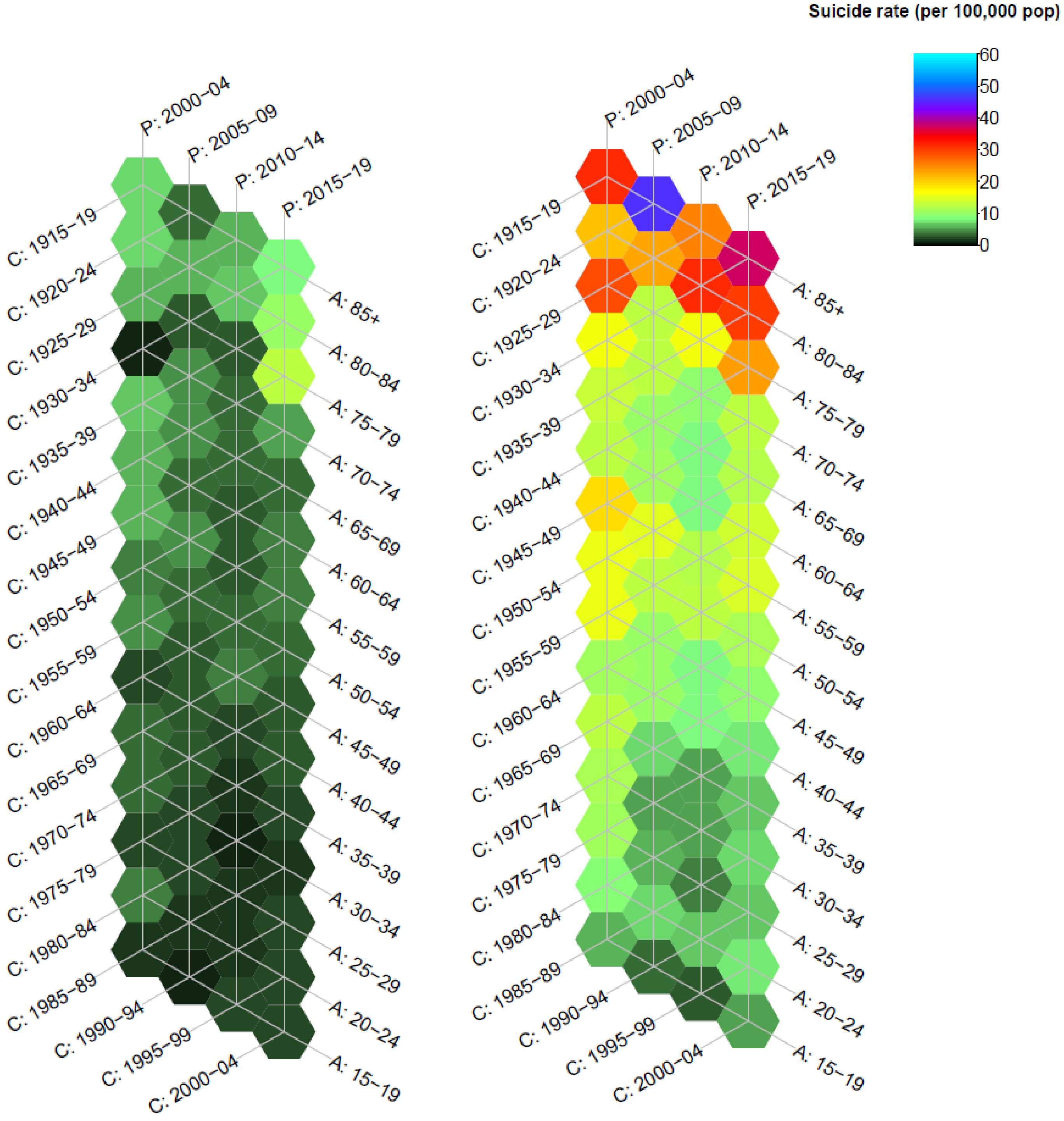

Supplementary Figure S5. Age, period, and cohort effects on suicide among native-born males and females between 2000-2019 in Spain (multi-phase method). A: Men. B: Women.

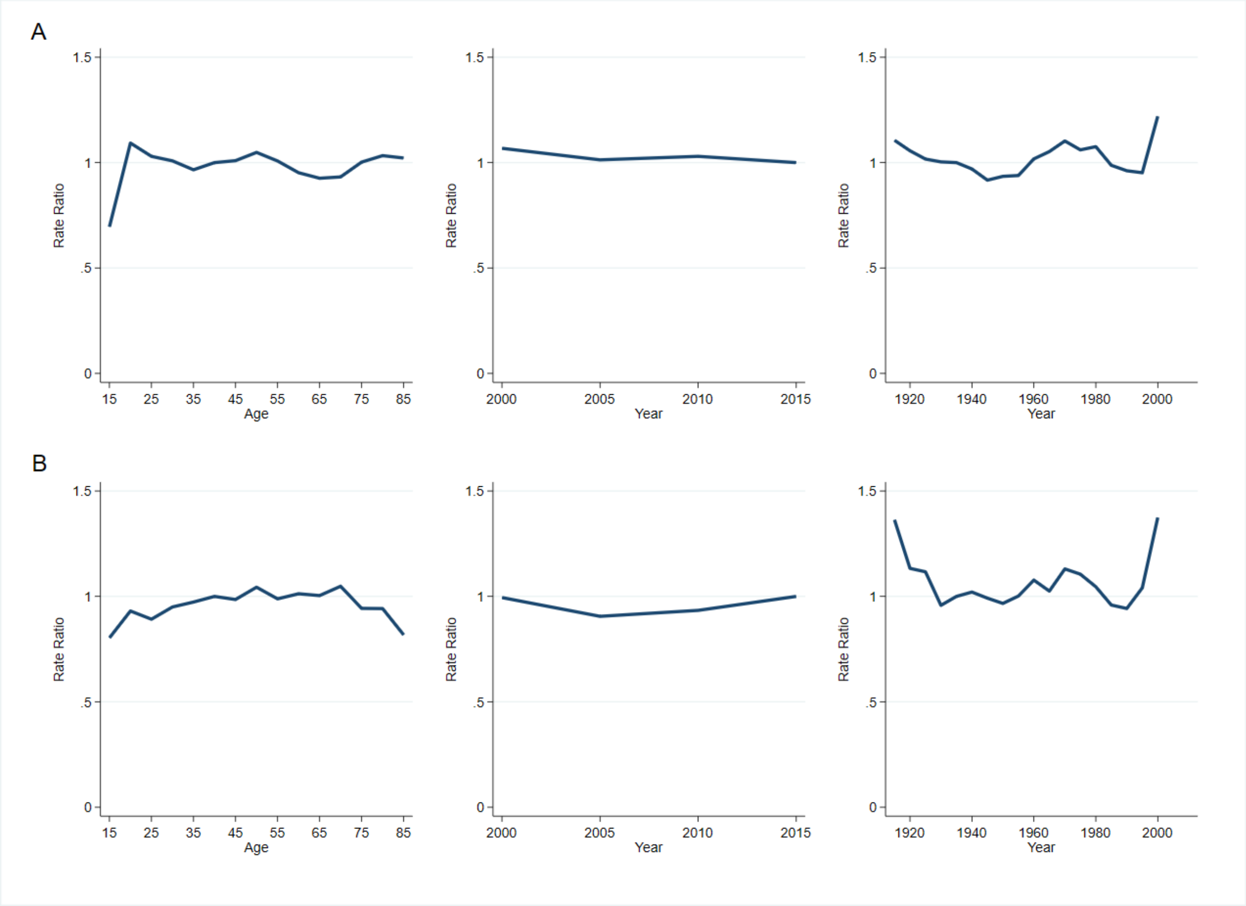

Supplementary Figure S6. Age, period, and cohort effects on suicide among foreign-born males and females between 2000-2019 in Spain (multi-phase method). A: Men. B: Women.

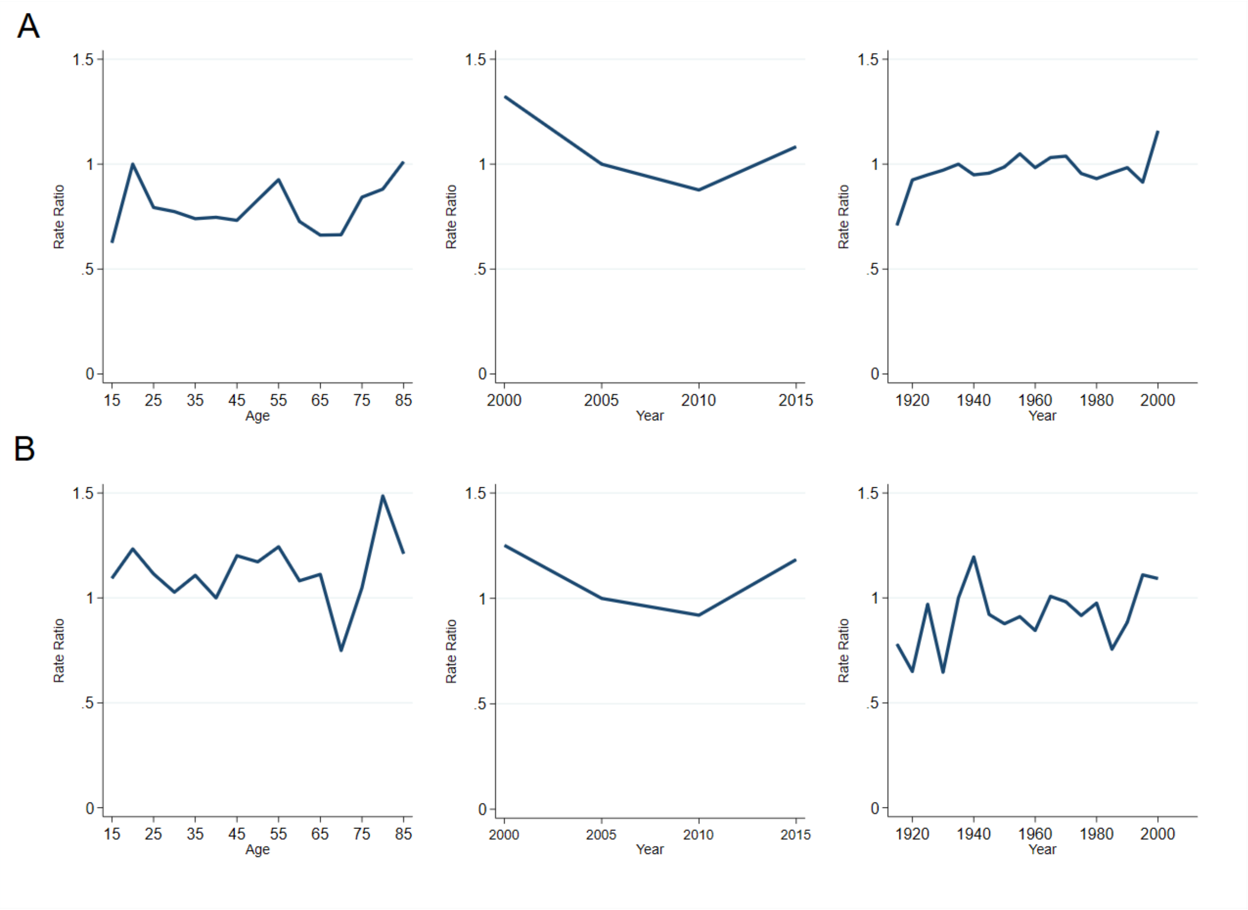

Supplementary Figure S7. Age, period, and cohort effects on suicide among native-born males and females between 2000-2019 in Spain (reference year: 1935).

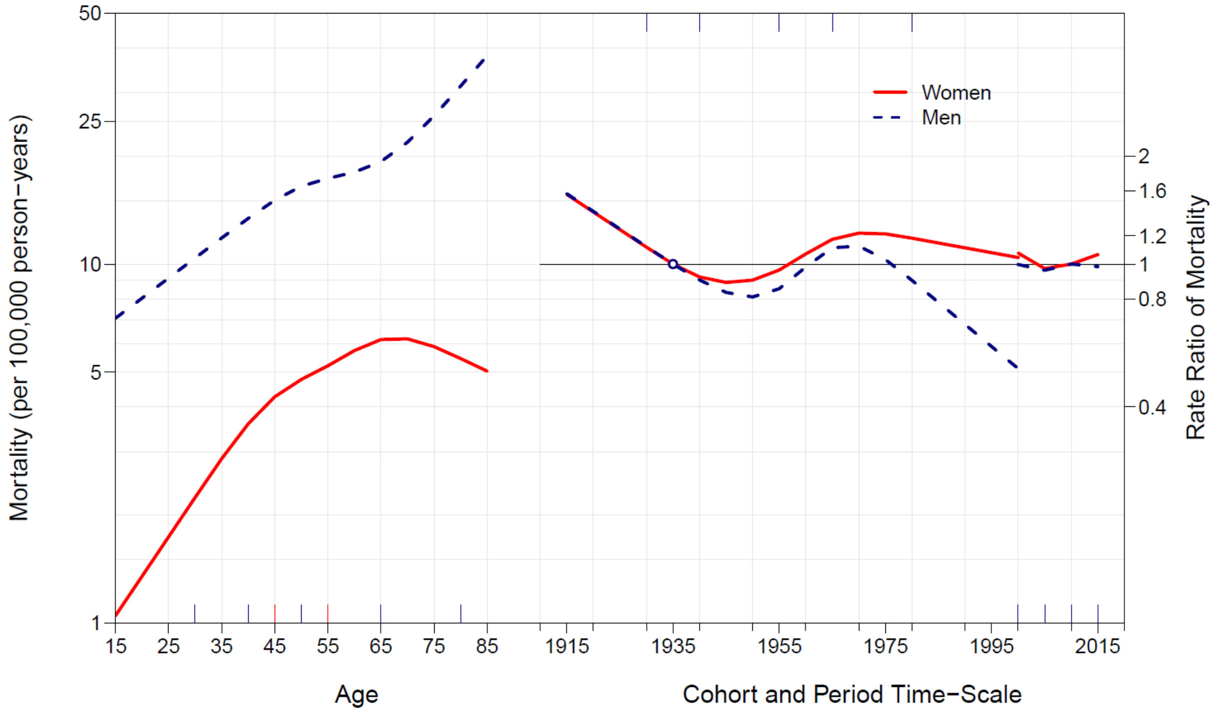

Supplementary Figure S8. Age, period, and cohort effects on suicide among foreign-born males and females between 2000-2019 in Spain (reference year: 1935).

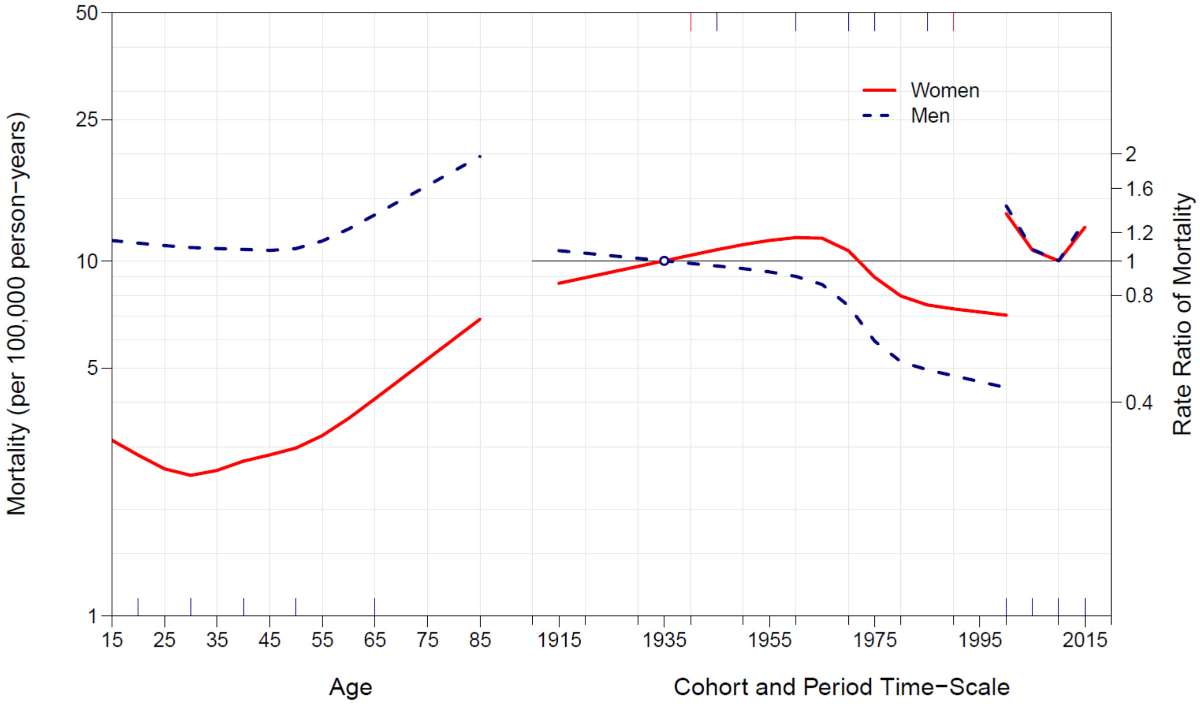

Supplementary Figure S9. Age, period, and cohort effects on suicide among foreign-born individuals between 2000-2019 in Spain, stratified by Spanish citizenship status (reference year: 1935).

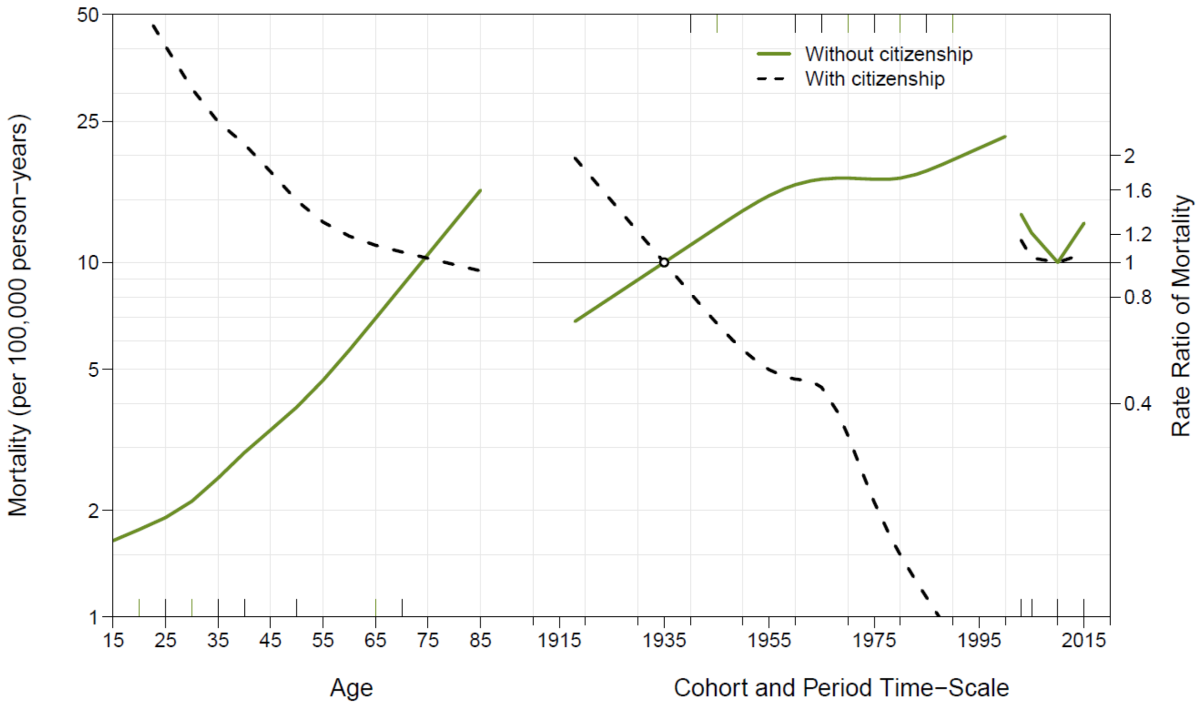

Supplement: Supplementary file 1 [file DataSheet1.PDF]
